# Does exposure to cigarette brands increase the likelihood of adolescent e-cigarette use? A cross-sectional study

**DOI:** 10.1136/bmjopen-2015-008734

**Published:** 2016-02-23

**Authors:** C Best, W van der Sluijs, F Haseen, D Eadie, M Stead, AM MacKintosh, J Pearce, C Tisch, A MacGregor, A Amos, M Miller, J Frank, S Haw

**Affiliations:** 1 School of Health Sciences, University of Stirling, Stirling, UK; 2 Child and Adolescent Health Research Unit, University of St Andrews, St Andrews, UK; 3 Institute for Social Marketing, School of Health Sciences, University of Stirling, Stirling, UK; 4 Centre for Research on Environment Society and Health, School of GeoSciences, University of Edinburgh, Edinburgh, UK; 5 ScotCen Social Research, Edinburgh, UK; 6 Usher Institute of Population Health Sciences and Informatics, School of Medicine, University of Edinburgh, Edinburgh, UK; 7 Public Health Research and Policy, The Usher Institute of Population Health Sciences and Informatics, College of Medicine and Veterinary Medicine, University of Edinburgh, Edinburgh, UK; 8 School of Health Sciences, University of Stirling, Stirling, UK

**Keywords:** PUBLIC HEALTH

## Abstract

**Objective:**

To examine the relationship between tobacco cigarette brand recognition, and e-cigarette use in adolescents.

**Design:**

Cross-sectional observational study.

**Setting:**

High schools in Scotland.

**Participants:**

Questionnaires were administered to pupils in Secondary 2 (S2 mean age: 14.0 years) and Secondary 4 (S4 mean age: 15.9 years) across 4 communities in Scotland. An 86% response rate with a total sample of 1404 pupils was achieved.

**Main outcome measures:**

Self-reported previous use of e-cigarettes and self-reported intention to try e-cigarettes in the next 6 months.

**Results:**

75% (1029/1377) of respondents had heard of e-cigarettes (69.5% S2, 81.1% S4), and of these, 17.3% (10.6% S2, 24.3% S4 n=1020) had ever tried an e-cigarette. 6.8% (3.7% S2, 10.0% S4 n=1019) reported that they intended to try an e-cigarette in the next 6 months. Recognition of more cigarette brands was associated with greater probability of previous e-cigarette use (OR 1.20, 99% CI 1.05 to 1.38) as was having a best friend who smoked (OR 3.17, 99% CI 1.42 to 7.09). Intention to try e-cigarettes was related to higher cigarette brand recognition (OR 1.41, 99% CI 1.07 to 1.87), hanging around in the street or park more than once a week (OR 3.78, 99% CI 1.93 to 7.39) and living in areas of high tobacco retail density (OR 1.20, 99% CI 1.08 to 1.34). Never having smoked was a protective factor for both future intention to try, and past e-cigarette use (OR 0.07, 99% CI 0.02 to 0.25; and OR 0.10, 99% CI 0.07 to 0.16, respectively).

**Conclusions:**

Higher cigarette brand recognition was associated with increased probability of previous use and of intention to use e-cigarettes. The impact of tobacco control measures such as restricting point-of-sale displays on the uptake of e-cigarettes in young people should be evaluated.

**Strengths and limitations of this study**

- E-cigarette use among young people is increasing, and the nature and determinants of this process are of great interest to health professionals.
- This is the first study to look at environmental determinants of e-cigarette uptake in adolescents.
- The study has a high response rate (86%).
- The sample is not nationally representative, but the logistic regression models have been adjusted to account for the demographic profile of participants.

## Introduction

E-cigarettes represent a rapidly expanding market. In 2012, there were 250 e-cigarette brands and around 3600 flavours for sale globally, increasing to 466 brands and 7700 flavours in 2014.^1^ E-cigarettes were originally designed to mimic conventional cigarettes, but manufacturers have since diversified their appeal by introducing multiple shapes, colours and flavours. In 2012, ‘big tobacco’ entered the e-cigarette market, and there has subsequently been a sharp increase in the advertising and promotion of e-cigarette products. It is estimated that approximately £8 million was spent by Skycig, Vype, Gammuci and E-Lites on advertising in all media—press, television, radio, internet and outdoor—combined in 2013.^2^ Of these companies, only Gammuci is still independent of tobacco company involvement.

Striking similarities have been noted between the imagery employed to advertise e-cigarettes and those historically used to promote cigarettes.^3^ E-cigarette advertisements build on some of the iconic tobacco brand imagery that is now illegal in television and print media in the USA and the UK.^4^ ^5^ Tobacco brand imagery has also found a place in contemporary youth culture with a recent study finding that 22% of UK Top 40 YouTube music videos contained tobacco imagery and 4% tobacco brands.^6^ These videos are highly accessed and viewed by a large proportion of young people.^6^ Another study found 70% of tobacco-related YouTube videos contained images of tobacco brands.^7^ Against this background, advertisements for e-cigarettes communicate to young people that e-cigarettes are a safer form of smoking and are a method of smoking that is permitted everywhere.^8^ ^9^ That is, consumers can have the positive elements of smoking communicated by tobacco brands without the health or social costs. Therefore, the young people most susceptible to e-cigarette uptake will be those already convinced of the positive attributes of smoking through exposure to cigarette brands and advertising. There is empirical evidence that exposure to tobacco advertising impacts on e-cigarette uptake in young people.^10^ Agaku and Ayo-Yusuf found that young people who recalled seeing any type of protobacco advertising were more likely to have used e-cigarettes. In addition, they found a dose–response relationship between the number of sources of protobacco advertising that young people recalled, and the odds of e-cigarette experimentation. One of the sources of exposure measured was through point of sale in retail outlets. The timing of that study is important because it is based on the 2011 National Youth Tobacco Survey conducted before the expansion of e-cigarettes into grocery/convenience-type stores,^11^ therefore, recall of tobacco advertising or packaging in stores is unlikely to be solely a proxy for e-cigarette advertising/brand exposure in this location.

Cigarettes have strong brands that have taken a long time to build up. These are being undermined by recent public health interventions but, nevertheless, they still remain a strong presence in public consciousness.^12^ E-cigarettes, however, do not as yet have strong brand identities due to the relatively recent expansion of this market and on-going instability.^13^Therefore, tobacco cigarette brands remain the main point of reference for a positive smoking identity among young people. Tobacco brand awareness is known to influence transition to smoking. Does tobacco brand awareness also influence e-cigarette uptake by virtue of the association between the products? This is plausible given the conceptual similarity and overlap in commercial ownership between the two products. This issue is particularly relevant now, as many countries are considering implementing tobacco point-of-sale display bans, and/or plain packaging for cigarettes.^14^

Smoking rates among young people are falling,^15^ but in sharp contrast, e-cigarette use is rapidly increasing among adolescents.^16^ For this reason, the likely impact of tobacco point-of-sale bans, or plain packaging on e-cigarette use in young people, will be of relevance to future international public health policy. The objective of this paper is to examine whether cigarette brand recognition is related to e-cigarette uptake in adolescence, and the potential predictive power of other linked factors affecting exposure to cigarette brands, such as smoking status, frequency of visits to retail outlets, tobacco outlet density, smoking status of friends and family members, and frequency of unsupervised leisure time outside the home (while controlling for other personal factors such as age, sex, socioeconomic status and ethnicity). Unsupervised leisure time outside the home was included as it was hypothesised to moderate the effects of tobacco retail outlet density as a source of opportunity for exposure. Unsupervised leisure time outside the home is also potentially a measure of parental supervision. Parental supervision is known to be related to smoking initiation^17^ which may also play a role in e-cigarette initiation.

## Method

The data presented here were collected as part of an ongoing 6-year multimodal study designed to assess the impact of Scottish legislation banning point-of-sale tobacco advertising and marketing in retail outlets on young people's smoking-related attitudes and behaviours.^18^ Smoking attitudes and behaviour were assessed through a school-based survey conducted annually, beginning in 2013, in four purposively selected communities. Four schools were selected to reflect two levels of urbanisation (urban vs small town), and two levels of social deprivation (high vs medium/low).

To capture data on the rapidly changing e-cigarette market, questions about awareness, and intention to try e-cigarettes were added to the 2014 wave of the study (after the implementation of the ban in large supermarkets, but before implementation in small shops). The results reported here are based on data collected in February 2014.

Questionnaires were administered to pupils in Secondary 2 (S2; mean age: 13.96 years) and Secondary 4 (S4; mean age: 15.91 years) by teachers during class time under exam conditions. Ethical approval was obtained from the University of St Andrews, School of Medicine, Ethics Committee. Parental opt-out consent was obtained prior to pupils completing the survey. Pupils also provided active consent on the day of the survey.

## Survey questions and derivation of variables

### Demographic variables

Respondents were asked their gender and date of birth. Individual family material well-being was assessed through the Family Affluence Scale (FAS).^19^ The FAS consists of four questions (own bedroom, number of family cars, number of computers, number of family holidays abroad per year). The FAS raw scores were transformed though categorical principal component analysis into single dimensional scores that were then divided into tertiles of high, medium and low FAS.^20^ Missing data (n=43) were imputed at the median.

### Cigarette brand awareness, smoking and e-cigarette status and experience

Cigarette brand awareness was assessed through brand recognition of 13 cigarette brands including one ‘fake’ brand name. Pupils who stated that they recognised the fake brand were excluded from the analyses (n=80).

Smoking status was assessed with the question: ‘Have you ever smoked cigarettes, even if it is just one puff?’ to which they could respond ‘yes’ or ‘no’. Negative responses to this question were used as our variable for ‘never smoked’. Pupils who responded that they had tried smoking were then asked whether ‘they used to smoke but have given up’, ‘only tried once or twice’ or are ‘current smokers’. This variable was dichotomised to ‘current smokers’ versus ‘not current smokers including never smokers’.

Pupils were also asked whether their parents were smokers and whether their best friend smoked.

### Frequency of visits to tobacco retail outlets and unsupervised leisure activity outside the home

Frequency of visits to shops was assessed for: supermarkets; confectioners, tobacconist and newsagents; grocers or convenience stores; garage or petrol stations; fish-and-chip shops or other take-aways; and mobile ice cream and burger vans. These were the tobacco retail outlets most likely to be visited by young people. Pupils could choose from ‘every day’, ‘most days’, ‘about 2 or 3 times a week’, ‘about once a week’, ‘less than once a week’, ‘never’ or ‘don't know’ for their frequency of visit to each type of outlet.

Pupils were asked how often they hung around in the street or park, and could indicate that they did this ‘every day’, ‘most days’, ‘weekly’, ‘less often’, ‘never’ or ‘don't know’.

###  E-cigarette use

This was assessed by presenting respondents with the statement ‘An e-cigarette is a tube that looks similar to a normal cigarette. An e-cigarette may have a glowing tip and puffs a vapour that looks like smoke but unlike normal cigarettes, they don't burn tobacco’. Respondents were then asked whether they had heard of e-cigarettes and could respond ‘yes’, ‘no’ or ‘don't know’. Respondents who answered ‘yes’ to this question were then directed to the question ‘Which ONE of the following is closest to describing your experience of e-cigarettes?’, to which they could respond ‘I have never used them’, ‘I have tried them once or twice’, ‘I use them sometimes (more than once a month)’ or ‘I use them often (more than once a week)’. This variable was dichotomised to ‘ever tried’ versus ‘never tried’. The next question was ‘Do you think that you will try e-cigarettes in the next 6 months?’ to which they could reply ‘yes I do’, ‘no I don't’ or ‘don't know’. This response was also dichotomised to ‘yes’ versus ‘no’ or ‘don't know’.

### Retail outlet density

The Tobacco Retailers Register was used to create a dataset of proximity-weighted tobacco retail outlets per square kilometre (km^2^) for every postcode in Scotland.^20^ These were matched to pupils’ home postcodes to give an individual measure of tobacco outlet density within an 800 m radius of the geographical centroid. There were 360 missing or partially recorded pupil postcodes, thus, retail outlet density could only be derived for 74.4% of the sample.

## Analyses

Statistical analyses were conducted in Stata V.13. Logistic regression models were constructed using purposeful selection.^21^ Variables in the preliminary multivariable model found to be non-significant predictors by Wald value p>0.05 were dropped from the model. Variables were tested as nested models (with constant sample size) using the Wald test (p<0.05—variable retained).

The significance threshold was adjusted to p=0.01 because of the low proportion of positive outcomes in the sample.^22^ The final models employed robust variance estimation to account for clustering by school.

## Results

The response rate to the survey was 86% and a total sample of 1404 pupils was achieved out of a possible 1633. The sample profile is given in table 1.

**Table 1:** Sample description

Variable	Number (valid %)	Missing n
Gender (male)	721 (51.76)	11
White ethnic group	1285 (93.12)	24
Current smoker	71 (5.26)	53
Never smoked	1116 (81.16)	31
Family affluence scale		
Low	467 (33.26)	0
Medium	487 (34.69)	0
High	450 (32.05)	0
Parental smoking		
No	853 (62.22)	33
Yes	518 (37.78)	33
Best friend smokes		
Yes	153 (11.06)	21
No	1230 (88.94)	21

**Variable**	**Mean (SD)**	

Age in years	14.46 (1.03)	27
Brand recognition	3.11 (2.60)	145
Tobacco retail outlet density	3.98 (3.67)	360

Fifty-two per cent of the sample was male and 93.1% were of white ethnicity. Five per cent of the sample (1.7% S2, 9.5% S4) reported they were current smokers, and 81.2% had never smoked conventional cigarettes. The average number of cigarette brands recognised was 3.1.

## Visits to shops and unstructured leisure activity

The most frequently visited shops were newsagents, with most respondents saying they went on ‘most days’. The modal frequency for visits to supermarkets and convenience stores was around once a week. The majority of respondents also reported visiting garages and take-aways once a week. Mobile burger/ice cream vans were the least frequently visited. Fifty per cent of pupils reported never visiting mobile vans.

The modal frequency for hanging around in the street or park was ‘never’ with 37.2% (n=510) reporting they never did this; 4.8% (66) reported they hung round in street or park every day, 12.6% (173) most days 13.7% (188) weekly and 31.7% (434) less than once a week.

## Awareness of e-cigarettes

Seventy-five per cent of respondents (1029 of 1377 who answered this question) had heard of e-cigarettes. Older pupils were more likely to have heard of e-cigarettes than younger ones (Fisher's exact test p<0.001), with 69.5% of S2 pupils having heard of e-cigarettes compared with 81.1% of S4 pupils. There was no statistically significant difference between men and women, or between respondents with different levels of socioeconomic deprivation as measured by FAS in awareness of e-cigarettes.

## Ever trying e-cigarettes

In our sample, 176 (17.3%: 10.6% S2, 24.3% S4) of 1020 respondents had tried an e-cigarette. Table 2 below shows which of the variables had a significant bivariate relationship with having tried an e-cigarette.

**Table 2:** Variables with significant bivariate relationship to e-cigarette experience

Categorical independent variables	Design-based F		p Value
Visit newsagent	F(1.03, 3.09)=10.84		0.04
Hang around street/park	F(1, 3)=97.36		0.002
Current smoker	F(1, 3)=216.82		<0.001
Never smoked	F(1, 3)=352.1271		<0.001
Parental smoking	F(1, 3)=16.83		0.03
Best friend smoking	F(1, 3)=432.52		<0.001

**Continuous independent variables**	**Likelihood ratio χ^2^**	**OR**	**p Value**

Age in years	33.80	1.62	<0.001
Brand recognition	119.75	1.44	<0.001
Retail outlet density	4.48	1.05	0.03

These variables were entered by purposive selection into a multivariable logistic regression on ‘ever tried an e-cigarette’ (table 3). Model 2 in the table is adjusted for the demographic variables gender, age, FAS and ethnicity.

**Table 3:** Logistic regression on ‘ever tried an e-cigarette’

Variable	Model 1 OR (99% CI)	Model 2 OR (99% CI)
Never smoked	**0.12 (0.06 to 0.21)**	**0.10 (0.07 to 0.16)**
Ever smoked	**1**	**1**
Brand recognition	**1.20 (1.06 to 1.36)**	**1.20 (1.05 to 1.38)**
Best friend smoke		
Yes	**3.31 (1.45 to 7.54)**	**3.17 (1.42 to 7.09)**
No	**1**	**1**
Gender		
Male		1
Female		1.03 (0.47 to 2.25)
Family Affluence Scale		
1 low		1
2 medium		1.19 (0.72 to 1.98)
3 high		0.80 (0.24 to 2.71)
White ethnic group		1
Other ethnic group		1.87 (0.45 to 7.76)
Age in years		0.96 (0.73 to 1.25)

Model 1 unadjusted model n=892, Pearson χ^2^ goodness-of-fit=53.11, p=0.59. The pseudo R^2^ value is 0.34. Model 2 adjusted model n=884, Hosmer-Lemeshow goodness-of-fit=7.04, p=0.53. The pseudo R^2^ value 0.36. Bold: p<0.01.

As shown in table 3, in the unadjusted model (model 1), recognising more cigarette brands (OR 1.20, 99% CI 1.06 to 1.36), and having a best friend who smoked (OR 3.31, 99% CI 1.45 to 7.54), increased the likelihood of having tried e-cigarettes. For every additional brand of cigarette, respondents recognised there was a 20% increase in their odds of having tried e-cigarettes. Having never smoked significantly reduced the odds (OR 0.12, 99% CI 0.06 to 0.21).

In the adjusted model (model 2), the ORs for having never smoked (OR 0.10, 99% CI 0.07 to 0.16) brand recognition (OR 1.20, 99% CI 1.05 to 1.38) and having a best friend who smoked (OR 3.17, 99% CI 1.42 to 7.09) remained statistically significant.

## Intention to try e-cigarettes in the next 6 months

Sixty-nine respondents out of 1019 (6.8%: 3.7% S2, 10.0% S4) said they planned to try e-cigarettes in the next 6 months. Ninety-three (9.1%; 6.5% S2, 11.8% S4) did not know whether they would or would not.

Variables found to have a significant bivariate relationship with positive intention to try e-cigarettes (‘yes’ as opposed to ‘no’ or ‘don't know’ responses) at p<0.05 threshold are shown in table 4.

**Table 4:** Variables with significant bivariate relation to intention to try e-cigarettes

Categorical independent variables	F (design based)		p Value
Hang around street/park	F(1, 3)=46.07		0.007
Current smoker	F(1, 3)=75.98		0.003
Never smoked	F(1, 3)=183.28		<0.001
Visit newsagent	F(1.04, 3.12)=14.66		0.03
Visit take-away	F(1.69, 5.07)=8.20		0.03
Parental smoking	F(1, 3)=41.42		0.008
Best friend smoking	F(1, 3)=268.35		<0.001

**Continuous independent variables**	**Likelihood ratio χ^2^**	**OR**	**p Value**

Brand recognition			
Retail outlet density	50.05	1.40	<0.001
Age in year	4.03	1.07	0.03
	15.50	1.65	<0.001

The above variables were then entered into a multivariable logistic regression by purposive selection with ‘positive intention to try e-cigarettes’ as the binary dependent variable (table 5).

**Table 5:** Logistic regression on intention to try e-cigarettes in next 6 months

Variable	Model 3 OR (99% CI)	Model 4 OR (99% CI)
Never smoked	**0.09 (0.03 to 0.30)**	**0.07 (0.02 to 0.25)**
Ever smoked	**1**	**1**
Brand recognition	1.33 (0.98 to 1.81)	**1.41 (1.07 to 1.87)**
Tobacco outlet density	**1.17 (1.06 to1.29)**	**1.20 (1.08 to 1.34)**
Hanging round in the street		
≥1/week	**4.34 (1.99 to 9.46)**	**3.78 (1.93 to 7.39)**
<1/week	**1**	**1**
Best friend smokes		
No	**5.84 (1.94 to 17.57)**	**8.18 (2.73 to 24.55)**
Yes	**1**	**1**
Parent smokes		
No	**1**	**1**
Yes	**0.70 (0.55 to 0.89)**	0.94 (0.54 to 1.65)
Gender		
Male		**1**
Female		**0.49 (0.27 to 0.91)**
Family Affluence Scale		
Low		1
Medium		1.70 (0.48 to 5.96)
High		1.47 (0.36 to 5.95)
White		1
Other ethnic group		0.43 (0.02 to 9.81)
Age in years		**0.55 (0.37 to 0.81)**

Model 3 unadjusted main effects n=695, Hosmer-Lemeshow goodness-of-fit (10)=1.52, p=0.99. Pseudo R^2^ value is 0.51.

Model 4 adjusted final model n=689, Hosmer-Lemeshow goodness-of-fit (10)=3.04, p=0.93. Pseudo R^2^ value is 0.55.

Bold: p<0.01.

Positive predictors of intention to try e-cigarettes in the next 6 months in the unadjusted model (model 3) were having a best friend who smokes was a positive predictor of intention to try e-cigarettes in the next 6 months in the unadjusted model (model 3) (OR 5.84, 99% CI 1.94 to 17.57). Never having smoked cigarettes was a protective factor against intending to try e-cigarettes (OR 0.09, 99% CI 0.03 to 0.30) as was having a parent who smoked (OR 0.70, 99% CI 0.55 to 0.89).

Respondents who reported hanging around the streets ‘more than once a week’ had more than three times the odds of intending to try e-cigarettes than respondents who hung around in the streets less frequently (OR 4.34, 99% CI 1.99 to 9.46). The higher the tobacco retail outlet density of the respondent's home environment the higher the odds that they would intend to try an e-cigarette in the next 6 months (OR 1.17, 99% CI 1.06 to 1.29).

The addition of the demographic variables in the final adjusted model (model 4) meant that the ‘parental smoking status’ variable no longer reached statistical significance (OR 0.94, 99% CI 0.54 to 1.65), tobacco outlet density (OR 1.20, 99% CI 1.08 to 1.34), never smoking (OR 0.07, 99% CI 0.02 to 0.25), hanging around the street (OR 3.78, 99% CI 1.93 to 7.39), and best friend smoking status (OR 8.18, 99% CI 2.73 to 24.55), remain statistically significant in the model. In addition, brand recognition (OR 1.41, 99% CI 1.07 to 1.87) also became statistically significant. Thus, with every additional cigarette brand know, there was a 41% increase in the odds of intending to try e-cigarettes.

## Discussion

In our study, just over 17% (10.6% S2, 24.3% S4) of young people had tried an e-cigarette at least once. Cigarette brand recognition was associated with previous e-cigarette use, as was having a best friend who smoked tobacco. Having never tried smoking tobacco was a strong protective factor against e-cigarette experimentation.

The nationally representative Scottish schools adolescent lifestyle and substance use survey (SALSUS^23^) found 7% of 13-year-olds, and 17% of 15-year-olds in Scotland, had tried e-cigarettes. Our survey (discounting the filter question on whether they had heard of e-cigarettes) found marginally higher levels of e-cigarette experience with 11% of 13-year-olds, and 25% of 15-year-olds having tried e-cigarettes. However, SALSUS was conducted 1 year before our survey, and the differences are set against a rapidly changing environment for e-cigarettes and reports of increasing prevalence of e-cigarette use among young people.

Nearly 7% of our sample intended to try an e-cigarette in the next 6 months. Significant predictors of intention to try e-cigarettes in the adjusted model were cigarette brand recognition, having a best friend who smokes, frequently hanging around the streets, and living in an area of high tobacco retail density. The significance of brand recognition in the models (with ever smoking as a covariate) suggests that there may be a direct route from tobacco awareness to e-cigarettes that is mediated by brand awareness and not necessarily involving active smoking. Further research is required to examine what it is about recognising more cigarette brands that influences e-cigarette uptake.

Respondents hanging around in the street once a week or more had three times the odds of intending to try e-cigarettes relative to respondents hanging around the streets less than once a week. Hanging round the streets may indicate both low levels of adult supervision which has previously been linked to adolescent risk behaviours,^24^ and opportunity for exposure to tobacco point-of-sale displays in the local area.^25^

When the strongest predictor variables (including never smoking, best friend smoking, and brand recognition) were entered into the preliminary logistic regression models, the frequency of visits to retail outlets did not contribute any additional information to the prediction of e-cigarette use. This indicates that although, for example, regularly going to newsagents is related to intention to try e-cigarettes at the bivariate level, brand recognition better encompasses both regular exposure to point-of-sale advertising, and the individual's susceptibility to these influences.

Young people with a best friend who smoked tobacco had three times the odds of having tried an e-cigarette and eight times the odds of intending to try e-cigarettes than those who did not report having a best friend who smoked. Choi and Foster^26^ found similar results in young adults (20–28 years), and White *et al*^27^ in adolescents (14–15 years). In addition, Choi and Foster found that those with a friend who smoked were more likely to believe e-cigarettes are less addictive than tobacco cigarettes. The authors suggest that this is because information about e-cigarettes is spread through social networks. Another possible explanation for the association between having friends who smoke and e-cigarettes use in young people may be due to clustering of experimentation within social groups.

In our sample, the relative importance of previous smoking experience versus current smoking in the prediction of intention to try e-cigarettes suggests that serial experimentation may be the dominant mode of use. That is, those who had tried tobacco were also likely to try e-cigarettes. In younger cohorts, there is also evidence that e-cigarette experimenters have weaker antismoking intentions.^28^ This supports the interpretation that trying one nicotine delivery method increases the risk for trying others. In addition, the only study to have found previous tobacco use was *not* a risk factor for e-cigarette use included both family tobacco use and sensation seeking as control factors,^15^ suggesting that sensation seeking may account for much of the covariance between e-cigarette and tobacco use. To date, most UK studies have found experimentation with e-cigarettes is far more common than regular use in young people.^29^ However, the possibility of serial experimentation by sensation seekers introduces a new ‘double jeopardy’, whereby young people who have tried a nicotine product without necessarily becoming addicted, or becoming regular users, subject themselves to another round of exposure. This is important because e-cigarettes have a great potential for diversification with new delivery mechanisms, designs and flavours enabling the continual induction of novelty for this group of young people.

Experimentation with nicotine can increase risk of addiction, as tobacco dependence has been shown to develop rapidly after the onset of intermittent cigarette smoking.^30^ ^31^ Furthermore, young people are more susceptible to addiction than older adults, and possible biological mechanisms for this have been identified.^32^ Further research on the long-term relationship between e-cigarette use and nicotine addiction is required to clarify these relationships.

### Study limitations

A study limitation is that this sample was not nationally representative. However, the logistic regression models were adjusted for the demographic characteristics of respondents to indicate how these variables influence the tendency to use e-cigarettes. In addition, comparing the distribution of FAS raw scores between this study (8.8% low, 34.0% medium and 50.1% high FAS) and those of the nationally representative Scottish Health Behaviours of School-Aged Children^20^ survey conducted in 2010 (9% low, 35% medium, 53% high FAS), shows a very similar distribution in terms of family material well-being. The SALSUS 2013 survey^23^ reported unweighted proportions of 50% men and women identical to the distribution in the 2013 local authority census of S2 and S4 pupils in Scotland. In our study, there were 52% men which is not statistically different to the population proportion (binomial test p=0.20).

A second limitation is that we did not have a measure of e-cigarette brand awareness and e-cigarette use by friends and family. Therefore, we could not estimate how much of the effect of cigarette brand awareness is due to young people who are exposed to cigarette brands at home through parental smoking also being exposed to e-cigarettes through parental e-cigarette use.

Furthermore, we do not know to what extent cigarette brand awareness is acting as a proxy for exposure to e-cigarette point-of-sale exposure. To investigate this issue, we included an e-cigarette advertising recall variable in the models (see online supplementary tables S1–3). Including this variable does not eliminate the significant effect of tobacco brand awareness. Therefore, even when exposure to e-cigarette advertisements and exposure to cigarette brands in the home through parental smoking are controlled for, tobacco cigarette brand awareness is a predictor of both e-cigarette use and intention to use.

10.1136/bmjopen-2015-008734.supp1 Supplementary tables

An additional limitation is that it would have been preferable to combine the two smoking status variables into a single variable. However, comparing two smoking categories to a never smoker reference category reduced the numbers in each category too far for meaningful statistical analysis given the very low rates of current smoking reported in this sample.

The strengths of this study are that it is the first to examine environmental (tobacco retail outlet density) and personal level (demographic, brand recognition and tobacco use) measures as predictors of e-cigarette use in young people. We have found that exposure to tobacco point-of-sale displays and/or packaging increases the probability of previous use and future intention to use e-cigarettes. This has implications for tobacco control policy in that an important outcome of restricting tobacco point of sale displays could be a reduction in adolescent experimentation with e-cigarettes. Further research is required to clarify the mechanisms by which cigarette brand recognition and tobacco retail outlet density influence e-cigarette uptake.

Nationally and internationally, the picture is complicated by the different trajectories of tobacco and e-cigarette regulation. For example, in Scotland, all tobacco point-of-sale displays were banned as of April 2015,^33^ and displays are also banned in the rest of the UK.^34^ However, many nations have yet to consider such interventions. In countries with high levels of youth experimentation with e-cigarettes, the potential effect of point-of-sale display restrictions on adolescent e-cigarette uptake may have implications for their future public health policy.

In the UK, as in other EU member states, e-cigarette displays and advertising will remain unregulated until the implementation of the EU Tobacco Products Directive^35^ in May 2016. In the UK, this is potentially a window of opportunity for e-cigarette promotion. There are already examples of cigarette gantry shutters advertising Vivid and Nicolite e-products (eg, see http://www.forecourtshop.co.uk/nicocigs-to-supply-free-tobacco-gantry-solutions-to-independent-retailers/), and it is highly likely that other e-cigarette manufacturers will actively seek to fill the blank space left by tobacco point-of-sale displays. Further work is planned to examine the relative influence of e-cigarette point-of-sale display and tobacco brand awareness on e-cigarette and cigarette uptake among adolescents over time as these changes take place in order to further inform international policy development. To conclude, in order to inform policy discussions in other jurisdictions, it is important to monitor the influence of newly implemented point-of-sale legislation on e-cigarette use as well as youth tobacco use.

## References

[1] Zhu SH, Sun JY, Bonnevie E Four hundred and sixty brands of e-cigarettes and counting: implications for product regulation. Tob Control 2014;23 (Suppl 3):iii3–9. 10.1136/tobaccocontrol-2014-051670 24935895 PMC4078673

[2] Bauld L, Angus K, de Andrade M E-cigarette uptake and marketing. A report commissioned by Public Health About Public Health England London, 2014.

[3] De Andrade M, Hastings G, Angus K Promotion of electronic cigarettes: tobacco marketing reinvented? BMJ 2013;347:f7473 10.1136/bmj.f7473 24361526

[4] Jones WJ, Silvestri GA The Master Settlement Agreement and its impact on tobacco use 10 years later: lessons for physicians about health policy making. Chest 2010;137:692–700. 10.1378/chest.09-0982 20202950 PMC3021365

[5] Parliament of the United Kingdom. Tobacco Advertising and Promotion Act. London: Stationery Office, 2002.

[6] Cranwell J, Murray R, Lewis S Adolescents’ exposure to tobacco and alcohol content in YouTube music videos. Addiction 2015;110:703–11. 10.1111/add.12835 25516167 PMC4402034

[7] Elkin L, Thomson G, Wilson N Connecting world youth with tobacco brands: YouTube and the internet policy vacuum on Web 2.0. Tob Control 2010;19:361–6. 10.1136/tc.2010.035949 20739706

[8] Richardson A, Ganz O, Stalgaitis C Noncombustible tobacco product advertising: how companies are selling the new face of tobacco. Nicotine Tob Res 2014;16:606–14. 10.1093/ntr/ntt200 24379146

[9] Farrelly MC, Duke JC, Crankshaw EC A randomized trial of the effect of e-cigarette TV advertisements on intentions to use e-cigarettes. Am J Prev Med 2015;49:686–93. 10.1016/j.amepre.2015.05.010 26163170

[10] Agaku IT, Ayo-Yusuf OA The effect of exposure to pro-tobacco advertising on experimentation with emerging tobacco products among U.S. adolescents. Health Educ Behav 2014;41:275–80. 10.1177/1090198113511817 24347143

[11] Rose SW, Barker DC, D'Angelo H The availability of electronic cigarettes in U.S. retail outlets, 2012: results of two national studies. Tob Control 2014;23 (Suppl 3):iii10–16. 10.1136/tobaccocontrol-2013-051461 24935892 PMC4078712

[12] Hastings G, Angus K Forever cool: the influence of smoking imagery on young people. British Medical Association - Board of Science. Roycroft G (ed). British Medical Association, 2008. http://hdl.handle.net/1893/9328

[13] Bauld L, Angus K, de Andrade M E-cigarette uptake and marketing. London: BMA Board of Science, 2014.

[14] Scheffels J, Lavik R Out of sight, out of mind? Removal of point-of-sale tobacco displays in Norway. Tob Control 2013;22:e37–42. 10.1136/tobaccocontrol-2011-050341 22678299 PMC3731079

[15] Currie C, Zanotti C, Morgan A Social determinants of health and well-being among young people. Health Behaviour in School-aged Children (HBSC) study: international report from the 2009/2010 survey. Copenhagen: 2012 http://www.euro.who.int/__data/assets/pdf_file/0003/163857/Social-determinants-of-health-and-well-being-among-young-people.pdf?ua=1

[16] Dutra LM, Glantz SA Electronic cigarettes and conventional cigarette use among U.S. adolescents: a cross-sectional study. JAMA Pediatr 2014;168:610–17. 10.1001/jamapediatrics.2013.5488 24604023 PMC4142115

[17] Kim HH, Chun J Examining the effects of parental influence on adolescent smoking behaviors: a multilevel analysis of the Global School-Based Student Health Survey (2003–2011). Nicotine Tob Res 2015 10.1093/ntr/ntv172 26272211

[18] Haw S, Amos A, Eadie D Determining the impact of smoking point of sale legislation among youth (Display) study: a protocol for an evaluation of public health policy. BMC Public Health 2014;14:251 10.1186/1471-2458-14-251 24628879 PMC4004271

[19] Currie C, Molcho M, Boyce W Researching health inequalities in adolescents: the development of the Health Behaviour in School-Aged Children (HBSC) family affluence scale. Soc Sci Med 2008;66:1429–36. 10.1016/j.socscimed.2007.11.024 18179852

[20] Currie C, Levin K, Kirby J Health Behaviour in School-Aged Children: World Health Organization Collaborative Cross-National Study (HBSC) HBSC Scotland National Report. Edinburgh, 2011.

[21] Hosmer DW Jr, Lemeshow S, Sturdivant RX Applied Logistic Regression. Hoboken, NJ: John Wiley & Sons, Inc., 2013.

[22] Long JS Regression models for categorical and limited dependent variables (Advanced Quantitative Techniques in the Social Sciences). SAGE Publications, 2013.

[23] Black C, Murray L, Dodds B Scottish Schools Adolescent Lifestyle and Substance Use Survey (SALSUS). Edinburgh, 2014 (cited 9 Feb 2015). http://www.isdscotland.org/Health-Topics/Public-Health/Publications/2014-11-25/SALSUS_2013_Technical_Report.pdf?1

[24] Wen X, Shenassa ED Interaction between parenting and neighborhood quality on the risk of adolescent regular smoking. Nicotine Tob Res 2012;14:313–22. 10.1093/ntr/ntr215 22121244

[25] Shortt NK, Tisch C, Pearce J The density of tobacco retailers in home and school environments and relationship with adolescent smoking behaviours in Scotland. Tob Control 2016;25:75–82. 10.1136/tobaccocontrol-2013-051473 25370699 PMC4717363

[26] Choi K, Forster J Characteristics associated with awareness, perceptions, and use of electronic nicotine delivery systems among young US Midwestern adults. Am J Public Health 2013;103:556–61. 10.2105/AJPH.2012.300947 23327246 PMC3567225

[27] White J, Li J, Newcombe R Tripling use of electronic cigarettes among New Zealand adolescents between 2012 and 2014. J Adolescent Health 2015;56:522–8. 10.1016/j.jadohealth.2015.01.022 25907651

[28] Moore GF, Littlecott HJ, Moore L E-cigarette use and intentions to smoke among 10–11-year-old never-smokers in Wales. Tob Control 2014 10.1136/tobaccocontrol-2014-052011 PMC4789807 25535293

[29] Bauld L, MacKintosh AM, Ford A E-cigarette uptake amongst UK youth: experimentation, but little or no regular use in nonsmokers. Nic Tob Research 2016;18:102–3. 10.1093/ntr/ntv132 26250882

[30] DiFranza JR Development of symptoms of tobacco dependence in youths: 30 month follow up data from the DANDY study. Tob Control 2002;11:228–35. 10.1136/tc.11.3.228 12198274 PMC1759001

[31] Scragg R, Wellman RJ, Laugesen M Diminished autonomy over tobacco can appear with the first cigarettes. Addict Behav 2008;33:689–98. 10.1016/j.addbeh.2007.12.002 18207651

[32] Christensen MH, Ishibashi M, Nielsen ML Age-related changes in nicotine response of cholinergic and non-cholinergic laterodorsal tegmental neurons: implications for the heightened adolescent susceptibility to nicotine addiction. Neuropharmacology 2014;85:263–83. 10.1016/j.neuropharm.2014.05.010 24863041 PMC4358794

[33] Tobacco and Primary Medical Services (Scotland) Act 2010 http://www.legislation.gov.uk/asp/2010/3/pdfs/asp_20100003_en.pdf

[34] Healthy Lives, Healthy People: A Tobacco Control Plan for England, Department of Health, March 2011. https://www.gov.uk/government/uploads/system/uploads/attachment_data/file/213757/dh_124960.pdf

[35] Revision of the Tobacco Products Directive. European Commission, March 2014. http://ec.europa.eu/health/tobacco/products/revision/index_en.htm

