# Supplementary material for: Does exposure to cigarette brands increase the likelihood of adolescent e-cigarette use? A cross-sectional study
Source: BMJ Open. 2016 Feb 23;6(2):e008734. doi: 10.1136/bmjopen-2015-008734 (PMC4769402; doi:10.1136/bmjopen-2015-008734)
Supplement: Supplementary tables [file bmjopen-2015-008734supp_tables.pdf]

Supplementary tables

The Model 5 in the table below shows the effect of including parental smoking, best friend smoking and current smoking in the logistic regression on e-cigarette experience (extending Model 2 in Table 3 main paper). Model 6 shows the effect of introducing e-cigarette advertising recall (extending Model 2 Table 3 main paper).

Table 1 Logistic regression on e-cigarette experience with additional variables: parental and best friend smoking status (Model 5) or e-cigarette advertising recall (Model 6).

Variable	Model 5 OR (99% CI)	Model 6 OR (99% CI)
**Current smoker**	4.34 (0.96 to 19.55)	**6.23 (1.24 to 31.36)**
**Not current smoker**	1	**1**
**Never smoked**	**0.12 (0.06 to 0.26)**	**0.10 (0.05 to 0.22)**
**Ever smoked**	**1**	**1**
**Brand recognition**	**1.17 (1.02 to 1.35)**	**1.24 (1.10 to 1.40)**
**E-cigarette advertising recall**		1
**Yes**		0.88 (0.56 to 1.39)
**Gender male**	1	1
**female**	0.92 (0.38 to 2.22)	1.02 (0.42 to 2.47)
**Family Affluence Scale (1 low)**	1	1

**Family Affluence Scale (2 med)**	1.45 (1.03 to 2.03)	1.34 (0.69 to 2.59)
**Family Affluence Scale (3 high)**	0.96 (0.28 to 3.27)	0.75 (0.15 to 3.64)
**White ethnic group**	1	1
**Other ethnic group**	11.76 (0.47 to 6.64)	1.98 (0.41 to 9.58)
**Age in years**	0.96 (0.71 to 1.29)	0.99 (0.74 to 1.33)
**Parents smoke No**	1	
**Yes**	1.42 (0.59 to 3.43)	
**Best friend smokes yes**	2.08 (0.79 to 5.49)	
**No**	1	

Model 5 n=860 Pseudo R2 =0.35 Hosmer-Lemeshow chi2(10) = 5.61 p = 0.69

Model 6 n=870 Pseudo R2=0.36 Hosmer-Lemeshow chi2(10) = 10.72 p= 0.22

The Model 7 in table 7 below shows the effect of including parental smoking, best friend smoking and current smoking in the logistic regression on intention to try e-cigarettes (extending Model 4 in Table 5 in main paper). Model 6 shows the effect of introducing e-cigarette advertising recall (extending Model 4 Table 5 in main paper).

Table 2 Logistic regression on intention to try e-cigarettes with additional variables: parental and best friend smoking status (Model 7) or e-cigarette advertising recall (Model 8)

**Variable**	**Model 7 Odds ratio (99% CI)**	**Model 8 Odds ratio (99% CI)**
Never smoked	**0.07 (0.02 to 0.25)**	**0.03 (0.01 to 0.09)**
Ever smoked	**1**	**1**
Brand recognition	**1.41 (1.07 to 1.87)**	**1.45 (1.12 to 1.89)**
Tobacco outlet density	**1.20 (1.08 to1.34)**	**1.17 (1.02 to 1.33)**
Hanging round in the street ≥1/wk	**3.78 (1.93 to 7.39)**	**3.58 (1.82 to 7.06)**
Hanging round in the street<1/wk	**1**	**1**
E-cig advert recall –yes		2.44 (0.32 to 18.42)
No		1
Parental smoking: No	1	

Yes	0.94 (0.54 to 1.65)	
Best friend smoking	**8.18 (2.73 to 24.55)**	
Gender male	**1**	1
female	**0.49 (0.27 to 0.91)**	0.56 (0.38 to 0.84)
Family Affluence Scale (low)	**1**	1
Family Affluence Scale (med)	1.70 (0.48 to 5.96)	1.82 (0.64 to 5.16)
Family Affluence Scale (high)	1.47 (0.36 to 5.95)	**1.83 (1.21 to 2.78)**
White	1	1
Other ethnic group	0.43 (0.02 to 9.81)	0.83 (0.06 to 11.23)
Age in years	**0.55 (0.37 to 0.81)**	0.48 (0.16 to 1.46)

Model 7 n=689 Hosmer–Lemeshow goodness-of-fit (10) =3.04 p=.0.93 Pseudo R^2 value is 0.55. Model 8 n=698 Hosmer–Lemeshow goodness-of-fit (10)) = 2.56 p=0.96 Pseudo R^2 value is 0.49

Bold text:p<0.01

Table 3 Differences between sample in smallest analysis (model 7) and excluded cases.

	In Model 7<br>N=689	Not in model 7<br>n=570
Brand awareness<br>Mean (sd)	3.45 (2.66)	2.70 (2.47)
Never smoked<br>N (%)	560 (81.28%)	556 (81.05%)
Male<br>N(%)	301 (43.69%)	420 (59.66%)
FAS low<br>N (%)	224 (32.51%)	245 (34.27%)
FAS medium<br>N (%)	219 (31.79%)	247 (34.55%)
FAS high<br>N (%)	246 (35.70%)	223 (31.19%)
Other ethnic group	28 (4.06%)	67 (9.70%)
Age	14.56 (1.04)	14.36 (1.02)

The participants not included are more likely to be male, low family affluence and non-white ethnic group. Their missing status is mostly due to not reporting a postcode upon which the tobacco outlet density measure was based.
